# Morphology and evolutionary significance of phosphatic otoliths within the inner ears of cartilaginous fishes (Chondrichthyes)

**DOI:** 10.1186/s12862-019-1568-z

**Published:** 2019-12-30

**Authors:** Lisa Schnetz, Cathrin Pfaff, Eugen Libowitzky, Zerina Johanson, Rica Stepanek, Jürgen Kriwet

**Affiliations:** 1 University of Birmingham, School of Geography, Earth and Environmental Sciences, Birmingham, B15 2TT UK; 2 University of Vienna, Faculty of Earth Sciences, Geography and Astronomy, Institute of Palaeontology, Geozentrum, Althanstraße 14, 1090 Vienna, Austria; 3 University of Vienna, Faculty of Earth Sciences, Geography and Astronomy, Institute of Mineralogy and Crystallography, Geozentrum, Althanstraße 14, 1090 Vienna, Austria; 4 Department of Earth Sciences, Natural History Museum, London, SW7 5BD UK

**Keywords:** Ear stone, Skeletal labyrinth, Elasmobranchii, Batoidea, Selachii, Apatite, Gnathostomes, Otoconia, Calcium carbonate, Infrared spectroscopy

## Abstract

**Background:**

Chondrichthyans represent a monophyletic group of crown group gnathostomes and are central to our understanding of vertebrate evolution. Like all vertebrates, cartilaginous fishes evolved concretions of material within their inner ears to aid with equilibrium and balance detection. Up to now, these materials have been identified as calcium carbonate-bearing otoconia, which are small bio-crystals consisting of an inorganic mineral and a protein, or otoconial masses (aggregations of otoconia bound by an organic matrix), being significantly different in morphology compared to the singular, polycrystalline otolith structures of bony fishes, which are solidified bio-crystals forming stony masses. Reinvestigation of the morphological and chemical properties of these chondrichthyan otoconia revises our understanding of otolith composition and has implications on the evolution of these characters in both the gnathostome crown group, and cartilaginous fishes in particular.

**Results:**

Dissections of *Amblyraja radiata*, *Potamotrygon leopoldi*, and *Scyliorhinus canicula* revealed three pairs of singular polycrystalline otolith structures with a well-defined morphology within their inner ears, as observed in bony fishes. IR spectroscopy identified the material to be composed of carbonate/collagen-bearing apatite in all taxa. These findings contradict previous hypotheses suggesting these otoconial structures were composed of calcium carbonate in chondrichthyans. A phylogenetic mapping using 37 chondrichthyan taxa further showed that the acquisition of phosphatic otolith structures might be widespread within cartilaginous fishes.

**Conclusions:**

Differences in the size and shape of otoliths between taxa indicate a taxonomic signal within elasmobranchs. Otoliths made of carbonate/collagen-bearing apatite are reported for the first time in chondrichthyans. The intrinsic pathways to form singular, polycrystalline otoliths may represent the plesiomorphic condition for vertebrates but needs further testing. Likewise, the phosphatic composition of otoliths in early vertebrates such as cyclostomes and elasmobranchs is probably closely related to the lack of bony tissue in these groups, supporting a close relationship between skeletal tissue mineralization patterns and chemical otolith composition, underlined by physiological constraints.

## Background

Chondrichthyans, the cartilaginous fishes, play a central role in our understanding of vertebrate evolution. The deepest phylogenetic split of jawed vertebrates (gnathostomes) is the divergence between chondrichthyans and osteichthyans, the bony fishes [1] and the monophyly of both sister taxa is strongly supported by both morphological [e.g., 2] and molecular data [e.g., 3]. The transition from the jawless vertebrates to the derived gnathostome body plan was accompanied by major morphological innovations leading to the divergence and dominance of gnathostomes within the vertebrates [4–6]. All gnathostomes share common features of the otic system [7], which is composed of two parts comprising semicircular canals and an otolithic element (the utriculus in fishes), and two otolithic organs (= sacculus and lagena in fishes), respectively [8–10].

The inner ear and vestibular system of marine vertebrates contain crystalline biocomposites of calcium carbonate and phosphate phases, which exist either as small separate microparticles (otoconia or statoconia), as an otoconial mass representing loose aggregates of multiple mono-crystals, or forming rigid polycrystalline otoliths (statoliths, “ear-stones”) in actinopterygians [e.g., 11]. These otoliths consist of agglutinated crystals or crystals precipitated around a nucleus that continuously grow forming concentric layers of organic matrix alternating with mineralized layers [12–14]. Nevertheless, both otoconia and otoliths can occur simultaneously in aquatic vertebrates [11].

Of these, only the otoliths of teleosts have been used in a wide range of scientific research, from age determination to species identification as well as (palaeo-) environmental interpretations [15–19]. Teleosts uniquely develop three pairs of otoliths within the inner ears, termed sagitta, lapillus and asteriscus, with the sagitta and lapillus being considerably larger than the asteriscus [20, 21]. The term otolith is also used for semi-rigid “ear-stones” in the inner ear of lampreys, which may have a single, amorphous otolith formed by fusion of spherical otoconia [22, 23]. Otherwise, extant agnathans have more or less spherical otoconia [8]. Semi-rigid otoliths also occur in extant holocephalans, some elasmobranchs and sarcopterygians, in which they are known to easily disintegrate indicating a very loose agglutination [13, 24–26]. Furthermore, otoliths also have been reported in an extinct lamprey [27] and in various numbers from one to three pairs in acanthiform acanthodians [13, 14, acanthodians representing stem chondrichthyans, e.g., 28]. Otoconia, conversely, have been found in all other major living jawed vertebrate groups, including cartilaginous fishes, and various interpretations of the phylogenetic significance of this character have been proposed [8, 29–33]. Both otoconia and otoliths are primarily used in equilibrium and balance detection, aiding the body to orient in all dimensions [34–36].

Additionally to semi-rigid otoliths and smaller otoconia, living chondrichthyans also possess a unique feature among all vertebrates with respect to these structures as they can incorporate exogenous material such as siliceous sand grains or various carbonate polymorph particles through the open endolymphatic duct duct located in the posterodorsal braincase as well as produce endogenous material within their inner ears [e.g., 37, 38]. Endolymphatic pores also were reported from a single acanthodian specimen, which indicates that this acanthodian also might have had both endo- and exogenous material in its inner ear [see e.g., 14, 39].

Chemical composition of the otoconia and otoliths has been uniformly reported to consist of four calcium carbonate polymorphs (calcite, vaterite, aragonite or calcium carbonate monohydrate) in extant gnathostomes [40–43]. In acanthodians, the otoliths consist of calcite [13, 44–47]. By comparison, the otoconia and amorphous otoliths in extant jawless agnathans consist of calcium phosphate in the form of apatite [8, 24, 48]. While Carlström [8], Ross & Pote [49] and Maisey [50] among others identified a phylogenetic pattern in the change of the chemical composition in vertebrate “ear-stones” from apatite in agnathans to calcite in gnathostomes, others such as Fermin et al. [23] and Schultze [13] assumed it to be the result of parallel evolution. Other hypotheses relate the differences in chemical composition to the ambient water chemistry [42] or the presence/absence of bony tissues [23]: The acquisition of mineralized bone (absent in living agnathans) is accompanied by the creation of regulatory systems that influence these tissues (e.g., resorption) such as humoral factors (transported by the circulatory system). Exchanging calcium phosphate for calcium carbonate may prevent these factors from affecting the otolithic system without having to invest in an additional system of homeostatic balance.

**Fig. 1:**
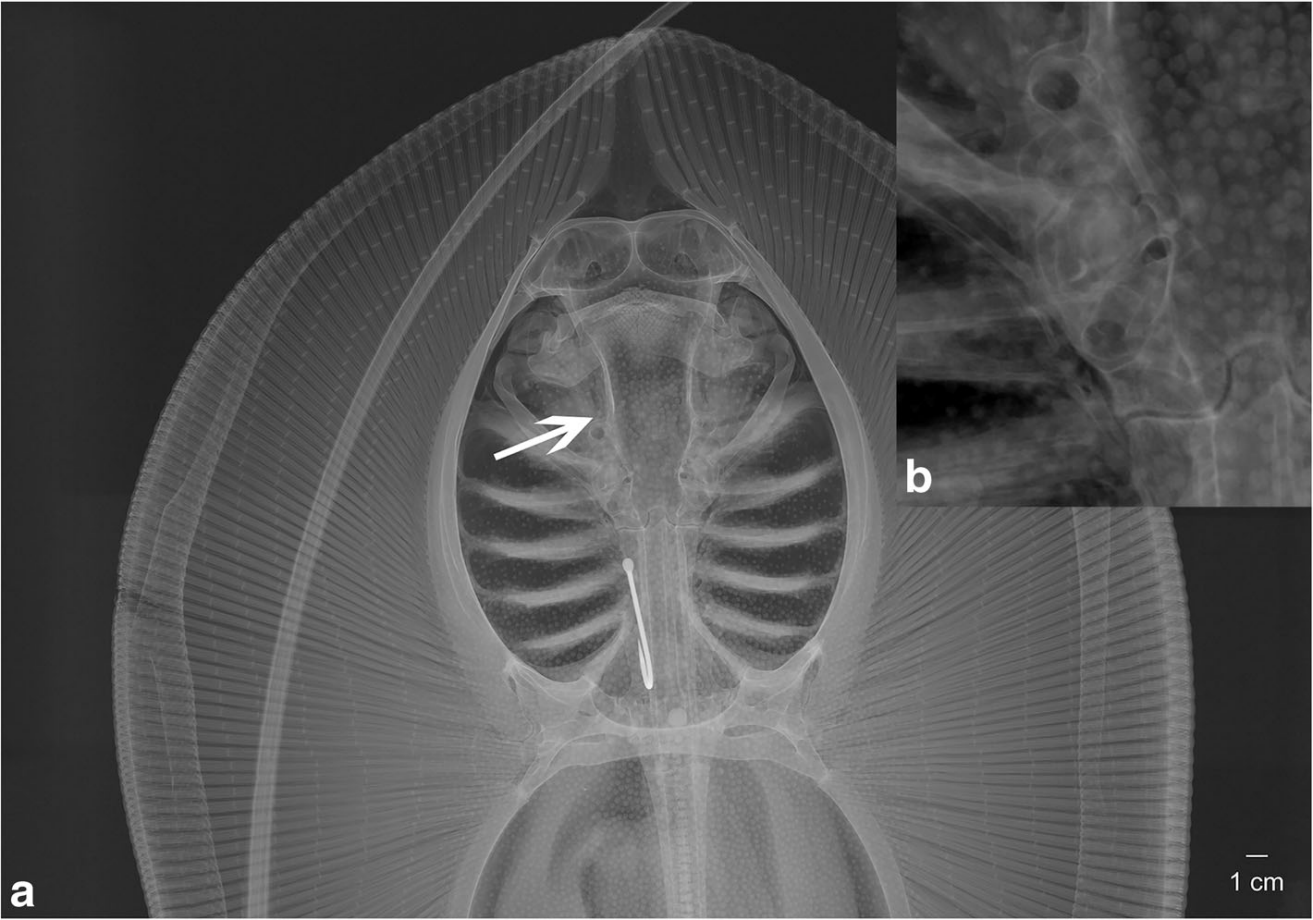
Radiograph of a female *Himantura signifer* (ZSM 30351) with the otolith structure (**a**, arrow). **b**) shows a close-up of the inner ear region

To identify possible phylogenetic or physiological signals in otolith development, we analysed crystalline biocomposites in elasmobranch fishes (sharks, skates, rays). For this purpose, we used micro-computed tomography (CT) scanning and X-ray imaging (Fig. 1) of 87 chondrichthyan individuals; three dimensional (3D) reconstruction software was applied to visualize the structures and their positions within the respective sensory organs. We found a series of structures within the inner ear, which do not match the descriptions of otoconia but are here instead identified as otoliths. To avoid confusion over terminology, we here nominate a polycrystalline mass, formed by the aggregation of multiple mono-crystals, with a distinctive morphology as otolith. Otoconia are defined as small separate microparticles, occurring in greater numbers than otoliths but considerably smaller and lacking the distinctive morphology of otoliths. We reinvestigated the morphological and chemical properties of the calcium-bearing structures within the skeletal labyrinth of the marine ray *Amblyraja radiata* and the freshwater ray *Potamotrygon leopoldi* as well as the small-spotted catshark, *Scyliorhinus canicula*. Dissections and infrared spectroscopy analysis were applied to describe the morphological details and measure the chemical compounds of the singular, polycrystalline otoliths in these individuals.

## Results

### Otolith positioning and morphology

**Fig. 2:**
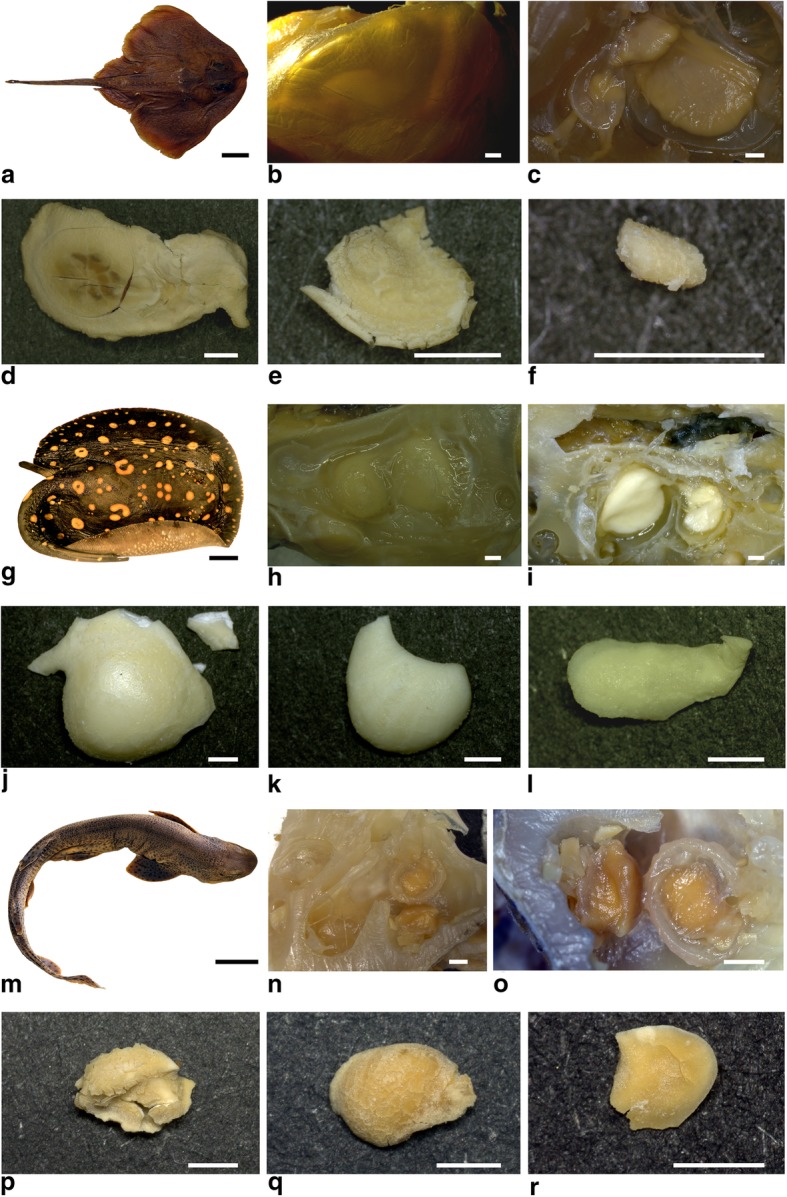
Section pictures of a female *Amlyraja radiata* (IPUW 7859) (**a-f**), a male *Potamotrygon leopoldi* (**g-l**) (IPUW 7358) and a male *Scyliorhinus canicula* (**m-r**) (EMRG-Chond-A-1). **b**) shows the posterior/anterior/horizontal semicircular canals in-situ, **c**) the skeletal labyrinth chamber with otoliths in-situ, **d**) sagitta **e**) lapillus **f**) asteriscus, **h**) the skeletal labyrinth chamber with the otoliths in-situ, **i**) the connection between sagitta and lapillus, **j**) sagitta, **k**) lapillus, **l**) asteriscus, **n**) the skeletal labyrinth chamber with otoliths in-situ, **o**) the sagitta and lapillus with the surrounding membrane-like structure, **p**) sagitta, **q**) lapillus, and **r**) asteriscus. A, G, M scale bar = 5 cm; B-F, H-L, and N-R scale bar = 1 mm

During dissection, we found three well-defined otolithic structures in the vestibular cavities of the inner ears of all investigated specimens and extracted these structures from both left and right vestibula of the rays *Amblyraja radiata and Potamotrygon leopoldi*, and the shark *Scyliorhinus canicula*, described here for the first time (Fig. 2). All elements developed some level of disaggregation when retrieved from the vestibular system and dried instantly upon air exposure. The three otoliths have different sizes, as in actinopterygians, and we apply the corresponding terminology. The largest otolith, which corresponds to the sagitta, is situated in the saccular chamber and a second, slightly smaller second otolith, interpreted as lapillus, occurs in the utriculus of each skeletal labyrinth in all specimens (Fig. 2c, i, n). A third, rather small otolith, the asteriscus, is present and although its position could not be fully resolved it is considered to be associated with the lagenar chamber. All three otoliths were separated by membrane-like structures (Fig. 2c, h, o). Irregular and unevenly spaced growth rings were found in the otoliths in *Amblyraja* (Fig. 2d-f), but not in *Potamotrygon* and *Scyliorhinus*.

**Fig. 3:**
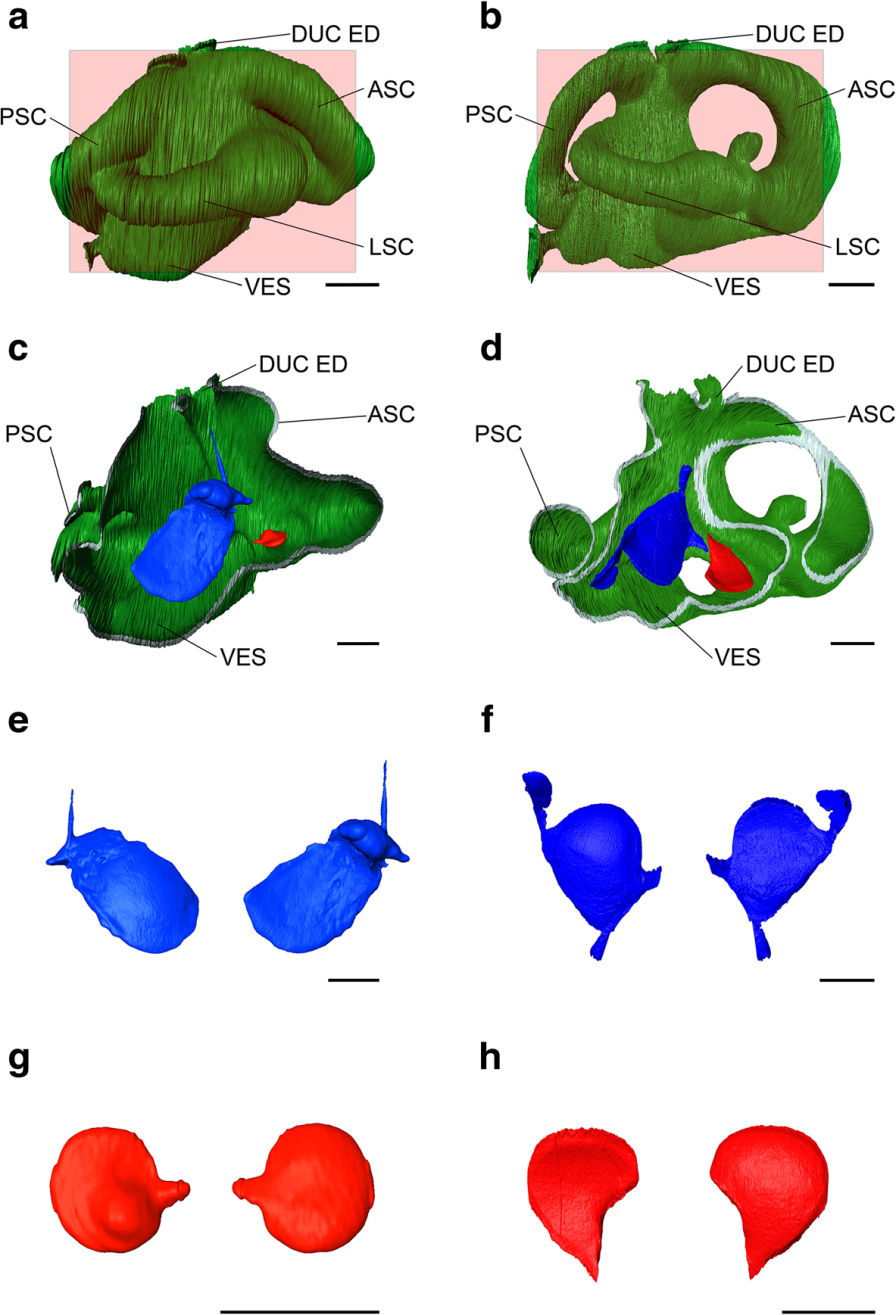
Virtual three-dimensional reconstruction of the left skeletal labyrinth and the sagitta and lapillus of *Amblyraja radiata* and *Potamotrygon leopoldi*. (**a**) Skeletal labyrinth reconstruction, (**c**) positioning of the otoliths within the skeletal labyrinth, (**e**) reconstruction of the sagitta, (**g**) reconstruction of the lapillus in *A. radiata*. (**b**) Skeletal labyrinth reconstruction, (**d**) positioning of the otoliths within the skeletal labyrinth, (**f**) reconstruction of the sagitta, (**h**) reconstruction of the lapillus in *P. leopoldi*. Scale bar = 2.5 mm. Red rectangles indicate plane of slicing. ASC, anterior semicircular canal; DUC ED, endolymphatic duct; LSC, lateral semicircular canal; PSC, posterior semicircular canal; VES, vestibulum

*Amblyraja radiata* exhibits a well-developed sagitta, elongated and thin. The inner face is concave while the outer face is convex. An extension protrudes into the endolymphatic duct in the 3D reconstruction (Fig. 3c). This structure, however, was not detected during the dissection but may have been inadvertently sliced away. On the dorsal margin, a prominent bulge is detected in both the reconstruction and the dried otolith (Figs. 2, 3d, e). Whether or not this represents a sulcus similar to the one in bony fish cannot be ascertained with certainty due to modification upon air exposure. The lapillus displays a rounded shape with a single extension protruding caudally (Fig. 2, 3e, g). It is flattened except for a bulge on the dorsal margin, which is otherwise slightly concave. The asteriscus is very small in size and elongated in length (Fig. 2f). Its morphology is difficult to assess as the structure proved to be very fragile after removal from the inner ear. Further morphological characters are not distinguished in this specimen.

The otoliths of *Potamotrygon leopoldi* display a considerably different morphology compared to *Amblyraja radiata*. The sagitta is elongated and rather round with a cranial extension (Figs. 2, 3j, f). The sagitta exhibits a very prominent sulcus located almost horizontally over the entire inner face. The lapillus is rounded throughout except for a convex curvature around the posterior margin (Figs. 2, 3k, h). In the 3D reconstruction, a slight dorsal extension is visible that probably was damaged during dissection and not found on the dried otolith. The asteriscus of *P. leopoldi* also is elongated and showed a slight curving with a small extension on one side (Fig. 2l). It was not detected in the micro CT scans and its exact position within the skeletal labyrinth was, therefore, difficult to assess.

The otoliths of the shark *Scyliorhinus canicula* differ considerably regarding their morphology. The sagitta is compact and rounded (Fig. 2p). There is a prominent bulge on the upper part, followed by a ridge. The edges of the sagitta are rather coarse and a fissure in the lower part of the inner side protrudes ventrally and to the sides. The lapillus is slightly elongated and ventrally rounded (Fig. 2q). It is concave and does not show any additional characteristics regarding its morphology. The asteriscus is round and exhibited a gentle curvature throughout the entire surface (Fig. 2r).

All specimens showed considerable differences regarding otolith morphology and shape. Especially, size differences between the lapilli of *A. radiata* and the two other species are obvious both in situ and after removal, while the sagitta of *A. radiata*, *P. leopoldi* and *S. canicula* are similar in their positioning within the skeletal labyrinth. *Scyliorhinus canicula* otoliths, however, were considerably smaller than in both rays. Exposure to air showed a stronger impact on the otolith structures of the marine *A. radiata* and *S. canicula*, whereas the otoliths of the freshwater *P. leopoldi* remained more or less perfectly intact.

### Infrared spectroscopy

**Fig. 4:**
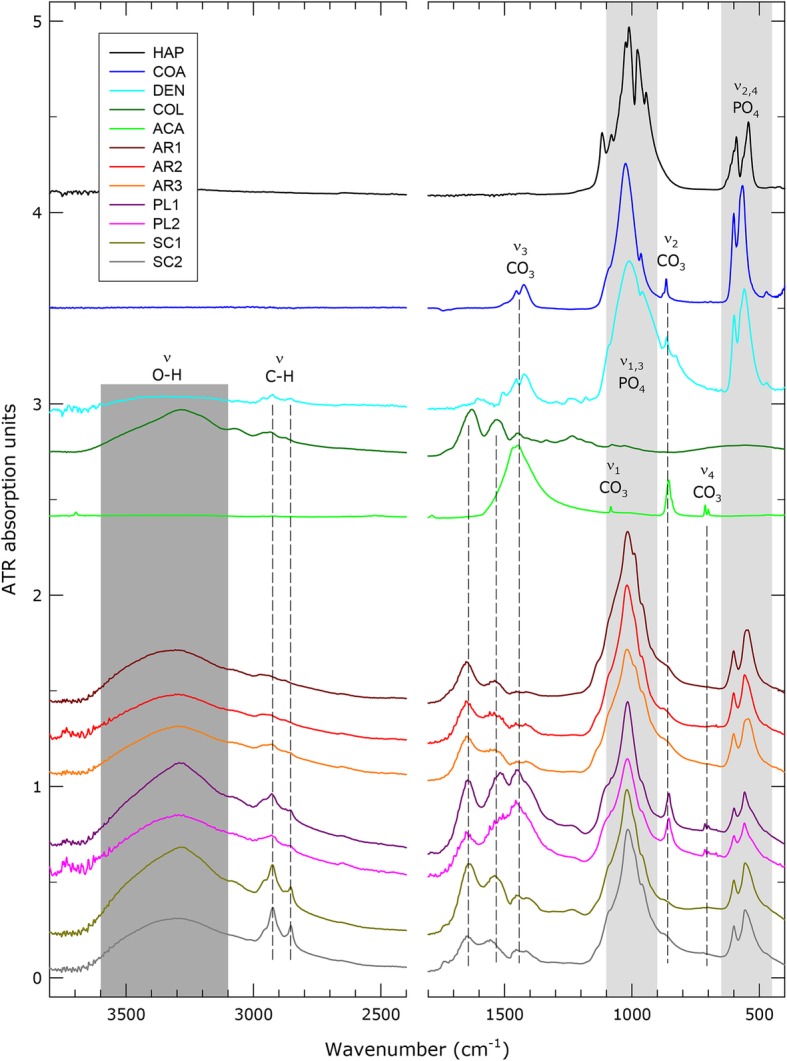
FTIR-ATR spectra of samples in comparison to reference material. Spectra have been vertically offset for better visibility. The region at 2400–1850 cm^− 1^ has been skipped due to numerous artefacts from CO_2_ and ATR-crystal defects. ACA, aragonite; AR1, *A. radiata* (IPUW 7858) sagitta; AR2, *A. radiata* (IPUW 7859) sagitta; AR3, *A. radiata* (IPUW 7859) asteriscus; COA, CO_3_-bearing apatite; COL, shark collagen; DEN, shark dentine + enamel; HAP, hydroxylapatite; PL1, *P. leopoldi* (IPUW 7358) lapillus; PL2, *P. leopoldi* (IPUW 7358) asteriscus; SC1, *S. canicula* (EMRG-Chond-A-1) sagitta, SC2, *S. canicula* (EMRG-Chond-A-1) lapillus

Infrared spectroscopy was performed to identify the chemical composition of the otoliths found in *P. leopoldi*, *A. radiata,* and *S. canicula*. Fourier-transform infrared attenuated total reflectance (FTIR-ATR) spectra of all seven samples (AR1 [*A. radiata*, IPUW 7858], AR2 [*A. radiata*, IPUW 7859], AR3 [*A. radiata*, IPUW 7859]; PL1 [*P. leopoldi*, IPUW 7358], PL2 [*P. leopoldi,* IPUW 7358], SC1 [*S. canicula*, EMRG-Chond-A-1] and SC2 [*S. canicula*, EMRG-Chond-A-1] and five reference spectra for comparison (HAP [hydroxylapatite], COA [CO3-bearing apatite], DEN [shark dentine + enamel], COL [shark collagen] and ACA [aragonite]) were analyzed (Fig. 4). In general, the spectra contain several regions that are characteristic for various vibrations. The area between ~ 2400 and 1800 cm^− 1^ has been omitted in Fig. 4, because it was corrupted by numerous artefacts from atmospheric CO_2_ and defects of the ATR-diamond crystal. The region between ~ 3600 and 3100 cm^− 1^ is assigned to the O-H stretching vibrations ν_1,3_ of water, i.e. moisture content of the samples, or (if sharp bands appear) hydroxyl groups in the structure of apatite. C-H stretching vibrations of organic material, e.g., collagen, usually appear at ~ 3100–2850 cm^− 1^. The section between ~ 1700 and 1000 cm^− 1^ shows a characteristic band pattern of collagen (unpublished observations). The band at ~ 1650 cm^− 1^ may contain also a weak component of the bending vibration ν_2_ of water molecules. The antisymmetric stretching vibrations ν_3_ of the carbonate groups in apatite, typically at 1450 and 1425 cm^− 1^ (unpublished observations) and the symmetric bending vibration ν_2_ are well visible. In addition, the weak carbonate ν_1_ (symmetric stretching vibration – inactive in calcite) and split ν_4_ (antisymmetric bending vibration) bands are visible in aragonite and in samples PL1 and PL2. The phosphate tetrahedrons display their infrared (IR) bands at ~ 1100–900 cm^− 1^ for the stretching vibrations ν_1,3_ and at ~ 650–450 cm^− 1^ for the bending modes ν_2,4_. Comparison of sample and reference spectra undoubtedly confirm the presence of carbonate/collagen-bearing apatite material in all samples. AR1–3 and SC1–2 are very similar, except that the bands from organic components are stronger in the latter two. In addition, there is strong evidence (split ν_4_ bands at ~ 700/712 cm^− 1^) for an aragonite component in samples PL1 and PL2.

### Phylogenetic framework

**Fig. 5:**
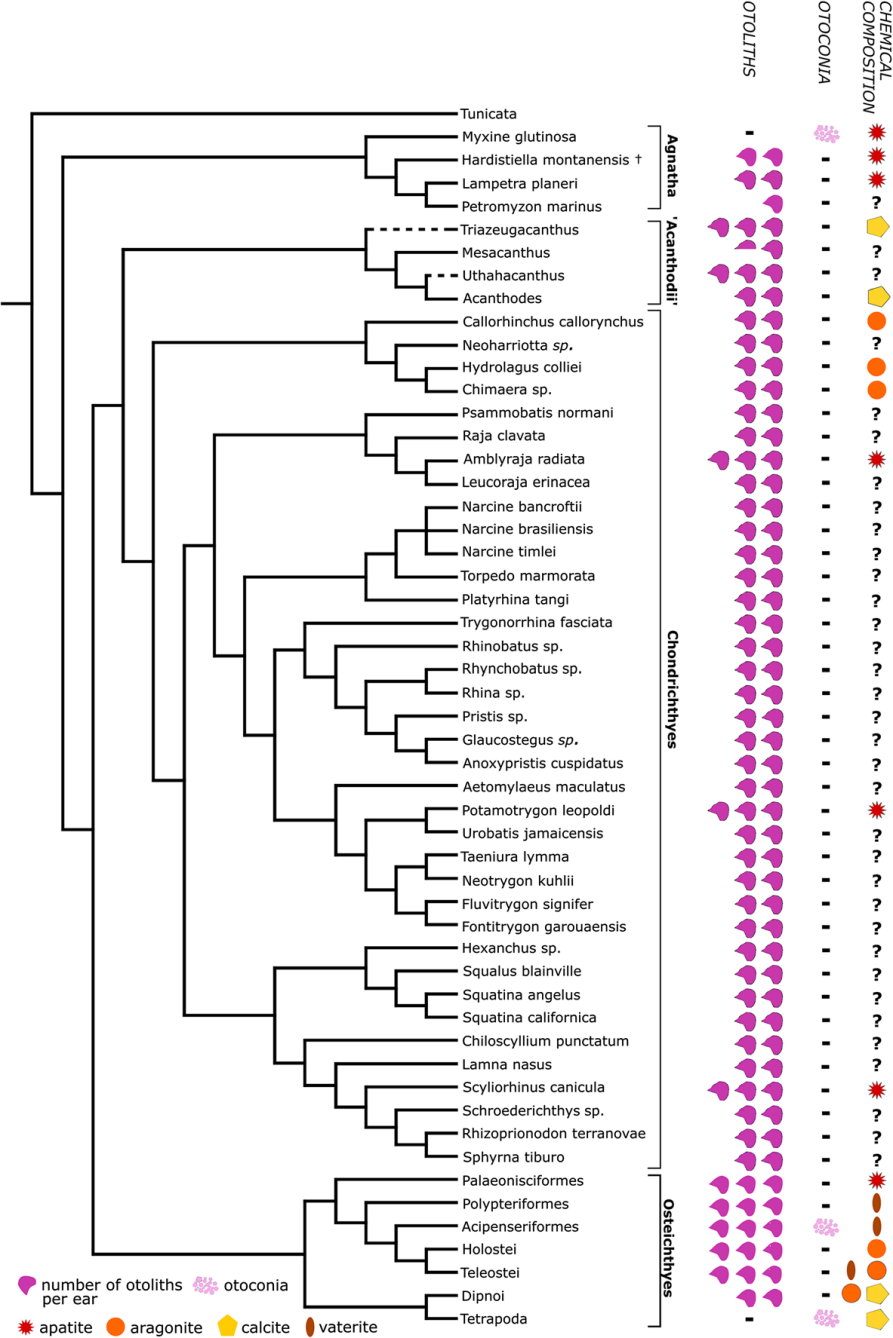
Distribution of otolith/otoconia and their chemical compositions on a phyologenetic tree depicting all major vertebrate groups, with a finer resolution of chondrichthyans. Phylogeny was composited from overall vertebrate data [51], acanthodian distribution [52], basal actinopterygians [53] and sarcopterygians [54] and extant chondrichthyan data [55, 56]. Otolith morphology and composition for the other major vertebrate groups was taken from literature: Acanthodii [13, 44, 45, 47], Agnatha [8, 27, 48], Osteichthyes [8, 40, 41, 43, 57, 58]

We plotted the distribution and composition of otoliths based on our investigations of 89 chondrichthyan individuals (combining information from both CT scans and dissections) on a phylogenetic tree of vertebrates, with a finer resolution of Chondrichthyes in particular (Fig. 5, Additional file 1). Data on the other major vertebrate groups was taken from published sources and Figcombined with the data from our analysis resulting in information for otolith structures and occurrences in 37 different species of cartilaginous fishes, thus providing the most detailed information about such structures in chondrichthyans to date. The evolutionary relationships between taxa were based on a composite tree drawn from published molecular and morphological data on vertebrates in general [51], recent data on Acanthodii [52], recent phylogenetic analyses of basal actinopterygians [53] and sarcopterygians [54], and extant chondrichthyans [55, 56]. The distribution of three characters (morphology, number and composition of otoliths) was mapped onto the existing phylogeny to allow a depiction of the range of occurrences of these characters. Otolith structures are widespread among all major chondrichthyan clades and consist of two pairs in most taxa, not exceeding three pairs. Two pairs were found in specimens investigated using CT scans only, while three otoliths were recognized in specimens investigated in situ using dissections. A shift from calcium phosphate to calcium carbonate material was observed, coinciding with the transformation from agnathans to gnathostomes. Within chondrichthyans, a shift from calcitic otoliths in acanthodians (stem group members) to apatite otoliths in crown-chondrichthyans occurred.

## Discussion

For the first time, we describe in detail the presence and morphology of endogenous polycrystalline otolith structures within the chondrichthyan skeletal labyrinth and provide an IR spectroscopy analysis of their material composition in representatives of all three major groups within elasmobranchs, including sharks, skates and rays. Our results enable us to propose that some cartilaginous fishes develop three otolith structures in each inner ear similar to what is commonly found in bony fishes, including otoliths of different sizes. The composition, however, differs considerably, with the integration of phosphate instead of carbonate in the elasmobranchs investigated. Previous studies of otoconial structures within chondrichthyans have focused on their single-crystalline nature and putative carbonate based chemical composition to draw conclusions with regards to the evolution of otoliths in gnathostomes. A significant result of the present study is that cartilaginous fishes seemingly show a higher variability in the shape as well as the chemical composition of these structures, which may have implications on their evolution throughout vertebrates as discussed below.

The elasmobranch specimens here investigated contained otolith structures with a distinct morphology, which differed significantly between *A. radiata*, *P. leopoldi*, and *S. canicula*. Otoliths of *A. radiata* and *P. leopoldi* resembled each other more closely than either to *S. canicula*, especially regarding the positioning within the skeletal labyrinth and the size of the elements. This may indicate a taxonomic signal in the morphology between different elasmobranch clades. CT scans of additional *P. leopoldi* specimens confirmed a close resemblance of both the sagitta and lapillus within the species, supporting such a taxonomic signal.

### Morphology of otolith structures in chondrichthyans

Previous studies assumed that small crystals of different calcium carbonate polymorphs (otoconia) were embedded upon the sensory macula within the inner ear of both elasmobranchs [29–32, 37, 40, 59–61] and holocephalans [8, 22, 62]. Only a single investigation recorded two polycrystalline otolith structures in the elephant fish, *Callorhinchus milii*, and matched them to the sagitta and asteriscus of bony fish [63]. Another study reported otoliths in different ontogenetic stages of *Leucoraja erinacea* but did not elaborate any further on their morphology or composition [64]. Thus, our results contradict previous findings and raise the question of why large, polycrystalline otoliths have not been reported before with certainty in cartilaginous fishes. For example, other than the holocephalan study just mentioned, a study focusing on the inner ear morphology in elasmobranchs [65] clearly showed otoconial masses (that we identify as large polycrystalline otoliths here) within the otoconial organs but did not mention their morphology or their composition any further. Otolith structures in the ray *Raja clavata* and the shark *Squalus acanthias* may have been known for a long time, but either were mistakenly interpreted as a series of singular crystals [8, 24, 31, 40, 60, 66] or only vaguely described without showing the morphology in detail [32, 67].

One possible explanation for this is related to the fragile composition of chondrichthyan otoliths when exposed to air. Such decomposition of otoliths into smaller, crystalline units also was observed in the current study; we assume that the binding forces between organic compounds and calcium phosphate crystals are weak compared to those in teleosts. The observed differences in preservation upon exposure to air between the marine *A. radiata* and *S. canicula* and the freshwater *P. leopoldi* may also indicate an environmental influence on these binding forces. A previous study also showed differences in the microchemistry of otolith structures (strontium;calcium ratios) in three different stingray species, which were linked to the different environments they inhabited [68]. Additional analyses of the organic compounds binding the apatite crystals therefore are necessary to understand whether the nature of these materials leads to an early fragmentation of otoliths in cartilaginous fishes, which may result in different interpretations from those made previously, but which are beyond the scope of this study. This could also have implications on the suitability of the morphology of these characters in taxonomic investigations, as the use of these is well-established in bony fishes [e.g., 15, 69–71].

### Chemical composition

In the past, chondrichthyan otoconial structures have been reported to be composed of either one of four calcium carbonate polymorphs (calcite, vaterite, aragonite or calcium carbonate monohydrate) using X-ray powder diffraction analysis [8, 13, 31, 72]. The present study cannot confirm these findings as all marine *A. radiata*, freshwater *P. leopoldi* and marine *S. canicula* individuals investigated contained a carbonate/collagen-bearing apatite material without any exogenous components. In addition, our results strongly contradict previous investigations in *A. radiata*, which reported multiple, single-crystalline structures of pure aragonite [8]. A transformation of minerals from calcium phosphate to calcium carbonate or vice versa within an individual is considered very unlikely as it requires extreme conditions, presumably leading to the destruction of the material, and therefore is rejected [13]. One earlier study, however, correlated the chemical composition of otoconia in some sharks with changes in ontogeny, showing a gradual replacement (but not a transformation) of amorphous hydrous calcium phosphate with aragonite material [73].

A switch from apatite to calcium carbonate has been related to an improved inner ear function as well as an advantage regarding the homeostatic control of different biomineralization processes (to be able to regulate otolith and bone deposition independently) within the ear [8, 19, 50, 74]. However, the underlying reasons for an evolutionary shift from phosphate to carbonate otoconial structures between different chondrichthyan taxa, cartilaginous fishes and higher gnathostomes remain ambiguous and are beyond the scope of this work but may be established in future investigations. Nevertheless, the specimens investigated here do not represent juveniles but rather adults and thus do not contradict our interpretations.

### Evolutionary aspects

The morphology and size of the otoliths within the skeletal labyrinth changed during the evolution of vertebrates. Phosphatic, amorphous single-crystalline bodies characterize living cyclostomes in contrast to polycrystalline otoliths in teleosts and extinct lineages such as acanthodians, as well as the reversion to single-crystalline structures of first aragonite and secondly calcite in sarcopterygians [8, 75, 76]. Establishing the occurrence of polycrystalline otolith structures composed of apatite in chondrichthyans has several implications for the evolution of otoliths in vertebrates. In contrast to earlier assumptions [e.g., 8, 13], calcium carbonate cannot be regarded to constitute the only material in gnathostome otoliths and otoconia. Similarly, proposing the presence of otoconia to be the plesiomorphic state in gnathostomes whereas well-organized otoliths characterize teleosts, may not be as straightforward as previously thought.

In this study, we recovered two to three pairs of otoliths within the inner ears of a broad phylogenetic spectrum of cartilaginous fishes. The difference between two pairs identified in micro CT scans and three pairs from in situ investigations is likely linked to limitations in resolution of these without prior staining of the specific structures, or low mineralization. In addition to our results, two to three singular otoliths have been reported in acanthodians [13, 14, 44–47], which have recently been reinterpreted as stem chondrichthyans [e.g., 1, 28, 52]. The otoliths of acanthodians (as far as known) consist of calcite rather than the apatite we have identified in the elasmobranchs investigated here. In holocephalans, the otoliths have been reported to consist of aragonite [22, 72]. The otoliths in the majority of bony fishes consist of calcium carbonate, either in the form of vaterite in all acipenseriforms, most polypteriforms and at least one teleost, or aragonite in a single polypteriform, holosteans, most teleosts, and sarcopterygians with a reversal to calcite in homeothermic tetrapods [41, 72]. Interestingly, the otoliths of Devonian palaeonisciform fishes representing basal actinopterygians have been reported to be made of phosphate [= apatite; 75].

This short review emphasizes that the chemical composition of otoliths in both chondrichthyans and osteichthyans is not as homogenous as previously thought. The presence of phosphatic otoliths in extant and extinct agnathans [e.g., 27] suggests that this chemical composition might represent the plesiomorphic condition for vertebrates. However, it previously has been argued that the chemical composition of otoliths correlates with the presence or absence of bony tissues [23]. Remarkably, vertebrates with no bony or strongly reduced bony tissues such as agnathans and elasmobranchs display phosphatic otoliths, whereas vertebrates with ossified skeletons such as acanthodians and bony fishes have calcitic otoliths. The presence of a largely cartilaginous endoskeleton associated with carbonatic otoliths in acipenseriformes (vaterite in *Acipenser*, vaterite and aragonite in *Polypterus*) does not contradict our interpretation, because the lack of an ossified endoskeleton is considered secondary and the crystals of the otoliths, once deposited, are very resistant to metabolic changes except under extreme stress [58]. Moreover, the otoliths of *Acipenser* represent an otoconial mass rather than a solidified polycrystalline otolith as in other bony fishes [72] consisting of two different parts, a base with a blade-like unit, and an apex resembling an aggregate of fused concretions [23]. The purposed presence of phosphatic otoliths in Devonian paleonisciform actinopterygians [13, 57] also might be related to a reduced endoskeletal ossification but the chemical composition of these otoliths needs to be tested further before final conclusions can be made.

We follow previous hypotheses to assume that the development of endochondral and dermal bones (which are apatite-based) in fishes may have resulted in the evolution of an independent otolithic system (calcium carbonate-based) from the skeletal system. This presumably allowed them to avoid creating an additional system of internal homeostatic balance based on humoral factors associated with bone and mineralization [23]. It also has been shown that the presence of macromolecules such as glycoproteins and polylactosaminoglycan on or near the maculae influences mineralization of the otoliths [77]. This further supports the interpretation that the mode of skeletal tissue mineralization influences the chemical composition of otoliths. One additional advantage of evolving this independent otolithic system, was that changes of the inorganic components and corresponding organic components are assumed to have produced better physical properties of the otolithic apparatus [23].

## Conclusion

Dissection and detailed analyses of the otolith structures in the skeletal labyrinths of the elasmobranchs *A. radiata*, *P. leopoldi*, and *S. canicula* reveal the presence of three solid, polycrystalline otoliths within each species with well-defined morphologies, as observed in bony fishes and acanthodians. Differences in the size and shape of otoliths between the two rays and the single shark may indicate a taxonomic signal within elasmobranchs. Thus, it might be possible to discriminate cartilaginous fishes on different taxonomic levels based on morphological details of their otolith structures. However, micro-CT scanning of additional 81 elasmobranch taxa revealed only two otoliths in each auditory system. This indicates that the third, smallest otolith may not be well calcified and thus dissection seems the only method for identifying the correct number of otoliths in elasmobranchs as for now.

In addition, an IR spectroscopy analysis shows that the otolith material within these dissected elasmobranchs is composed of carbonate/collagen-bearing apatite, which has not been identified in chondrichthyans before.

The incorporation of phosphate rather than carbonate into otoliths may be the plesiomorphic condition for vertebrates and probably closely associated with the lack of bony tissues. Nevertheless, subsequent studies are required to test this further. The close relationships between skeletal tissue mineralization patterns and chemical otolith composition do however indicate underlying physiological constraints.

We were able to refute the general assumption of elasmobranchs lacking otoliths and having non-phosphatic otoconia as shown in previous studies. The new insights and results of our study open pathways for future investigations, both regarding morphological adaptations of otoliths in different chondrichthyan lineages as well as the significance and function of this character throughout vertebrate evolution.

## Methods

In total, 89 chondrichthyan taxa housed in the institutional collections of the University of Vienna, Austria, the Natural History Museum in London, UK, and additional information from the #ScanAllFish database [78] were analyzed to investigate the presence of otoliths (as defined here; see above) in different taxa of Chondrichthyes. In addition, two adult *Amblyraja radiata* (IPUW 7858, IPUW 7859), one adult *Potamotrygon leopoldi* (IPUW 7358) and one adult *Scyliorhinus canicula* (EMRG-Chond-A-1) from the palaeontological collection of the University of Vienna, Austria (abbreviations IPUW and EMRG), were examined regarding in-situ positioning of otoliths and material analysis. Specimens had been preserved in 75% ethanol in the collection. In addition, 81 taxa were analyzed non-invasively with the micro CT devices Skyscan/Bruker 1173 (University of Vienna, Vienna) and Nikon Metrology HMX ST 225 (Image and Analysis Centre, Natural History Museum, London), because it was not possible to dissect these institutional housed specimens. CT scans of six additional taxa were retrieved from the #ScanAllFish database [78]. Of the four dissection specimens, only IPUW 7859 and IPUW 7358 were CT scanned prior to dissection and are included in the 81 taxa analyzed non-invasively with the micro CT devices in Vienna and the National History Museum. IPUW 7858 and EMRG-Chond-A-1 were dissected only to infer otolith composition and morphology. For a detailed list of investigated taxa and respective CT parameters see Additional file.

The CT scans were visualized using the free software Drishti (ANU Vizlab, Canberra, Australia) and the Bruker software DataViewer v. 1.4.4 (SkyScan/ Bruker microCT, Kontich, Belgium). The inner ear regions of a total of 87 taxa were screened for the presence and number of composite otolith structures which were readily identifiable in the scans as brightly shaded, dense structures well separated from the rest of the inner ear cavity. The outlines of these otoliths were smooth and without any grainy structure and therefore conform to the expected morphology of otoliths. If the structures did not meet these criteria, they were not classified as otolith structures (see Additional file).

### Otolith positioning and morphological analyses

The skeletal labyrinth of selected specimens of *A. radiata* (IPUW 7859), *P. leopoldi* (IPUW 7358) and *S. canicula* (EMRG-Chond-A-1) were exposed by either dissecting the relevant area from the whole specimen or excising a tissue block including the skeletal labyrinth and part of the orbital cavity. Otoliths of a second specimen of *A. radiata* (IPUW 7858) were retrieved for material analysis only without information on exact positioning and morphology. Skin and adjacent tissues were removed until cartilage of the otic capsule was visible. Cartilage was then carefully sliced off until the position of the semicircular canals were located. The semicircular canals were subsequently removed and the vestibular region opened dorsolaterally. Positions of the otoliths within the inner ear were first documented, followed by the extraction of the structures. Before and during the preparation, the tissue was kept in 75% ethanol and the removed otoliths were air-dried prior to the material analysis. The descriptive terms sagitta, lapillus and asteriscus for the structures investigated were used based on the resemblance in positioning and morphology to teleosts. The third otolith of both species could not be reconstructed from the CT scans and was only visible upon dissection. Images of the in-situ position and the single otoliths were taken using a Keyence VHX-1000D 3D digital microscope. Images presented here were adjusted using Adobe Photoshop CS5 (version 12.0, Adobe Systems, San José, USA) concerning color balance, contrast, and labeling.

### 3D reconstruction

The micro CT data sets represented as tiff-stacks of one specimen of *A. radiata* (IPUW 7859) and one specimen of *P. leopoldi* (IPUW 7358) were further processed to create 3D reconstructions of the left skeletal labyrinths and the respective otoliths using Amira v. 5.4.1 (FEI Visualization Sciences Group, Oregon, USA). Structures were segmented manually using the brush tool only. For reconstructing the inner ears, every second image was labeled initially, with a subsequent interpolation to accelerate the process. Positions within the skeletal labyrinth reconstructions were obtained using the ‘ObliqueSlice-Tool’ to cut through the labyrinth. The results allowed a comparison of the quality of the 3D reconstructions with the in-situ structures.

### Infrared spectroscopy

An infrared spectroscopy analysis was carried out to identify the material composition of the otolithic structures found in *A. radiata*, *P. leopoldi,* and *S. canicula*. Air-dried samples of two *A. radiata* (IPUW 7858, IPUW 7859)*,* one *P. leopoldi* (IPUW 7358) and one *S. canicula* (EMRG-Chond-A-1) were analysed, resulting in a total of seven samples. FTIR powder spectra were acquired from 370 cm^− 1^ to 4000 cm^− 1^ on a Bruker Tensor 27 FTIR spectrometer equipped with a glo(w) bar MIR light source, a KBr beam splitter, and a DLaTGS detector. Sample and background spectra were averaged from 32 scans at 4 cm^− 1^ spectral resolution. The undiluted sample was crushed and pressed on the 2 × 3 mm diamond window of a Harrick MVP 2 diamond attenuated total reflectance (ATR) accessory. Background spectra were obtained from the empty ATR unit. Data handling was performed with OPUS 5.5 software (Bruker Optik GmbH, 2005). Five reference spectra for comparison were obtained at identical conditions from shark collagen and dentine + enamel (unpublished observations), and hydroxylapatite resulting from annealed chicken bone. In addition, spectra of the Raman Research Used For Fun (RRUFF) data base [79] were employed, i.e. aragonite R040078 from Aragon, Spain, and carbonate-bearing F-apatite R050529 from Limburg an der Lahn, Germany (~ 20% of the phosphate sites are substituted by carbonate groups). However, it must be emphasized that ATR spectra are always slightly red-shifted to lower wavenumbers in comparison to pure transmission spectra, contingent on ATR-crystal material and band intensity [80].

### Phylogenetic framework

Based on the inner ear investigations of 89 chondrichthyan individuals, a total of 37 species of chondrichthyans were found to exhibit otolith structures. Of these, three were investigated using both CT scanning as well as dissecting whereas the remaining 34 species were examined using the non-invasive CT method exclusively. We mapped the distribution and composition of otoliths in those 37 species onto a composite phylogenetic tree of vertebrates, combining our results from all X-ray imaging, CT scanning and IR spectroscopy. Information on otolith morphology and chemical composition of the other major vertebrate groups was taken from the literature and combined with the data from our analysis. The evolutionary relationships between taxa were based on a composite tree drawn from published molecular and morphological data on vertebrates [51], data on Acanthodii [52], phylogenetic analyses of basal actinopterygians [53] and sarcopterygians [54], and extant chondrichthyans [55, 56].

## Supplementary information

**Additional file 1.** Original excel dataset for this study, based on CT scans and dissections.

## Data Availability

The material and datasets used and analysed during the current study are available from the corresponding author on reasonable request.

## Abbreviations

3D: Three dimensional
ACA: Aragonite
AR 1: *A. radiata* (IPUW 7858) sagitta
AR2: *A. radiata* (IPUW 7859) sagitta
AR3: *A. radiata* (IPUW 7859) asteriscus
ASC: Anterior semicircular canal
ATR: Attenuated total reflectance
COA: CO3-bearing apatite
COL: Shark collagen
CT: Computed tomography
DEN: Shark dentine + enamel
DUC ED: Endolymphatic duct
EMRG: Evolutionary Morphology Research Group collection, Department of Palaeontology, University of Vienna
FTIR: Fourier-transform infrared
HAP: Hydroxylapatite
IPUW: Department of Palaeontology, University of Vienna
IR: Infrared
LSC: Lateral semicircular canal
PL1: *P. leopoldi* (IPUW 7358) lapillus
PL2: *P. leopoldi* (IPUW7358) asteriscus
PSC: Posterior semicircular canal
RRUFF: Raman Research Used For Fun
SC1: *S. canicula* (EMRG-Chond-A-1) sagitta
SC2: *S. canicula* (EMRG-Chond-A-1) lapillus
VES: Vestibulum
